# Thyroid gland metastasis arising from breast cancer: A case report

**DOI:** 10.3892/ol.2013.1287

**Published:** 2013-04-03

**Authors:** MEI YANG, WEI WANG, CHENFANG ZHANG

**Affiliations:** 1 Departments of General Surgery, General Hospital of Guangzhou Military Command of PLA, Guangzhou 510010, P.R. China; 2 Pathology, General Hospital of Guangzhou Military Command of PLA, Guangzhou 510010, P.R. China

**Keywords:** breast cancer, metastasis, thyroid gland

## Abstract

The thyroid gland is an uncommon site for metastasis to develop and thus metastases arising from breast cancer are rarely observed. In the present study, we describe a case of a 45-year-old female with a three-year history of breast cancer who presented with a thyroid mass that was diagnosed as metastatic breast carcinoma by histopathological analysis of the subtotal thyroidectomy specimen. To ascertain the diagnosis of metastatic breast cancer, we evaluated two types of markers; those that possessed a similar expression status in the original and metastatic lesions [ER, PR and CerbB-2 (HER2/neu)], and those that are capable of differentiating between metastatic lesions and the surrounding thyroid components (TG and TTF-1). The results showed that ER, PR and CerbB-2 demonstrated a similar expression pattern in primary breast carcinoma and thyroid lesions. Meanwhile, in the thyroid lesions, the malignant cells showed negative staining for TG and TTF-1, which confirmed that lesions were not thyroid in origin. This case may prompt clinicians that although thyroid gland are uncommon metastatic site, a diagnosis of metastatic disease should be considered when new aggregates are identified in the thyroid glands and histopathological analysis may aid the diagnosis.

## Introduction

The most common site of breast cancer metastasis is the bone. Other common metastatic sites include the lungs, pleura, liver and brain (1). The majority of metastatic lesions occur shortly after the detection of the primary tumor (2). Metastasis to the thyroid gland is rare (3). The thyroid gland is also a rare site for metastatic disease to develop (4,5) and metastatic lesions from non-thyroid cancers are infrequent. However, since treatment strategies used to control primary and metastatic malignancies are different, accurate diagnosis appears important and may influence the prognosis and likelihood of a cure for lesions of the thyroid. Therefore, when encountering a patient with thyroid tumor, and with a long history of breast cancer, clinicians should be aware of the possibility of metastatic disease, until such a diagnosis can be ruled out. Then, besides collecting full clinical information, further histological analysis should be performed. Here, we report a case of thyroid metastasis arising from breast cancer. The thyroid metastasis appeared 3 years after the initial diagnosis of breast carcinoma and was confirmed by histopathological analysis.

## Case report

### Clinical presentation and diagnosis

The patient, a 45-year-old female, was diagnosed with an infiltrating ductal carcinoma in the right breast (cT3N1M0) by fine needle aspiration biopsy in June 2009. The patient subsequently received six cycles of neoadjuvant chemotherapy. A follow-up mammectomy was performed in November 2009. In June 2012, the patient presented with palpable masses on the right thyroid, isthmus and lateral neck. Subsequently, the patient underwent a bilateral subtotal thyroidectomy and lymphadenectomy. Histological assessment of the surgical thyroid specimens and the neck lymph nodes revealed multiple carcinoma foci. The microscopic and immunohistochemical findings confirmed that the thyroid masses were breast cancer metastases.

The primary lesion excised from the right breast was a 20×18×15-mm infiltrating ductal carcinoma. The metastases excised from the right and left thyroid three years later were 30×20×15 and 20×15×12 mm in size, respectively, and the lymph nodes contained sheets of metastatic carcinoma (4/9). The diagnosis of metastatic carcinoma was made based on immunostaining data for the estrogen receptor (ER), progesterone receptor (PR) and CerbB-2 in neoplastic cells. Additionally, staining for thyroglobulin (TG) and thyroid transcription factor 1 (TTF-1), which are expressed in the thyroid gland but not in breast cancer tissue, was performed to differentiate the thyroid component from neoplastic breast cells.

**Figure 1:**
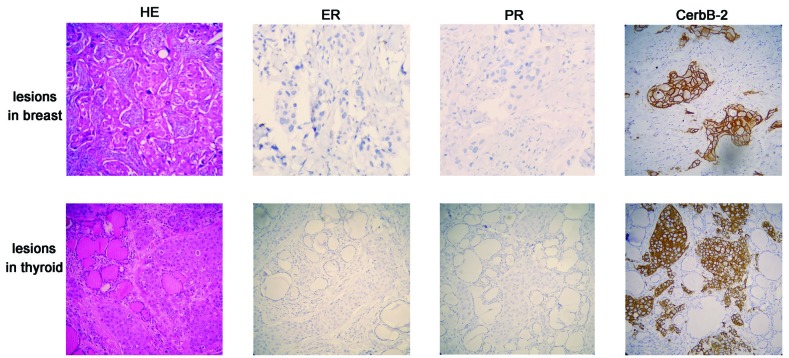
Hematoxylin and eosin (HE) staining and immunostaining (ER, PR and CerbB-2) of malignant lesions from the breast and thyroid gland. The malignant lesions from the breast and thyroid gland exhibited identical expression patterns for ER (negative), PR (negative) and CerbB-2 (strongly positive). Magnification, ×200. ER, estrogen receptor; PR, progesterone receptor.

**Figure 2:**
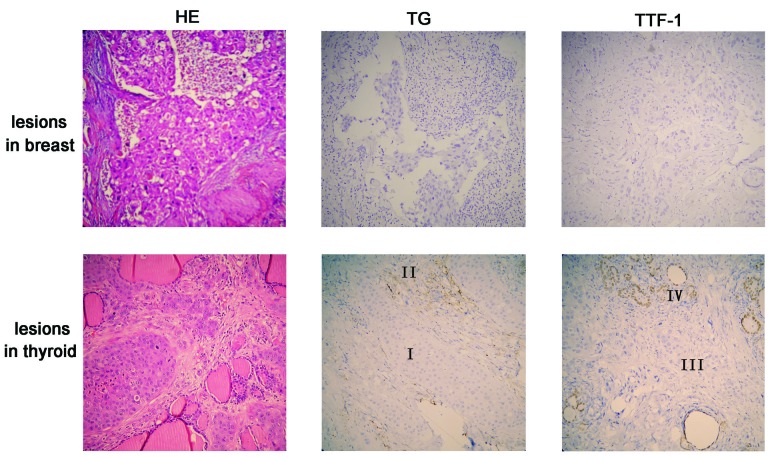
Hematoxylin and eosin (HE) staining and immunostaining (TG and TTF-1) of malignant lesions from the breast and thyroid gland. Upper panel: the malignant lesion and the adjacent breast component exhibited negative staining for TG and TTF-1. Lower panel: the normal component of the thyroid tissue was positive for TG (region II) and TTF-1 (region IV); however, metastatic cancer cells (regions I and III) were negative. Magnification, ×200. TG, thyroglobulin; TTF-1, thyroid transcription factor 1.

Histologically, pleomorphic tumor cells from the breast carcinoma metastasis were intermingled with the thyroid follicles (Fig. 1, hematoxylin and eosin staining of the adenocarcinoma tissue in the breast and thyroid gland). In the thyroid gland, clusters of metastatic breast carcinoma cells were surrounded by a normal thyroid component. Tumor cells in metastatic foci exhibited negative staining for the ER and PR and strong positive staining for CerbB-2, similar to the staining pattern of the primary breast lesion. The adjacent normal thyroid tissue was positive for TTF-1 (Fig. 2, lower panel, region IV) and TG (Fig. 2, lower panel, region II); however, the metastatic tumor cells were negative for both markers (Fig. 2, lower panel, regions III and I). These two markers indicate that the cancer lesions did not originate from the thyroid component.

## Discussion

This study reports the observation of an uncommon metastatic site (thyroid gland) of breast carcinoma in a patient with a three-year history of breast cancer. Clinically evident metastases of non-thyroid malignancies to the thyroid gland are uncommon and usually, patients with metastatic thyroid lesions present with similar symptoms to primary lesions. Particularly in breast cancer, metastasis to the thyroid gland is rarely observed. In addition, given the long interval between the primary cancer and subsequent thyroid metastases, diagnosis of thyroid metastasis from breast carcinoma may be difficult. However, importantly, the diagnosis influences the prognosis and likelihood of a cure for lesions of the thyroid gland, and the treatment strategy used to control metastatic breast carcinoma is different from that used to treat primary thyroid malignancies. Therefore, a diagnosis of metastatic disease should be considered when new aggregates are identified in the thyroid glands of patients with a long-term history of breast cancer, until such a diagnosis can be ruled out.

To ascertain metastatic breast carcinoma in the thyroid gland, immunohistochemistry should be performed for the detection of specific markers. There are at least two types of marker that are required to be evaluated; the markers which possess a similar expression status in original and metastatic lesions and those that are capable of differentiating between metastatic lesions and the surrounding thyroid components. Clinically, ER, PR and CerbB-2 (HER2/neu) statuses are known to be correlated with breast cancer. As they provide important therapeutic and prognostic markers in the management of breast carcinoma (6), they are routinely evaluated. In this study, immunohistochemical staining of the ER, PR and CerbB-2 in postoperative thy roid specimens of patients were performed and they all demonstrated a similar expression pattern with primary breast carcinoma; negative for ER and PR, and strongly positive for CerbB-2 (Fig. 1).

Furthermore, TG (7) and TTF-1 (8), the functional markers for medullary thyroid carcinoma, are useful to distinguish breast metastatic lesions from primary thyroid lesions. As shown in Fig. 2, normal thyroid follicular cells, but not malignant cells, are stained strongly for TG and TTF-1, which confirms that lesions are not thyroid in origin.

Based on the present study, we conclude that although knowledge of a patient’s complete clinical history is extremely useful, immunohistopathological analysis should be performed to confirm the diagnosis of metastatic breast cancer. Negative immunohistochemical staining for TG and TTF-1 in the thyroid lesions and similar staining results for ER, PR and CerbB-2 in breast and thyroid lesions may aid the diagnosis of thyroid carcinoma derived from metastatic breast cancer.
